# Global SUMO Proteome Responses Guide Gene Regulation, mRNA Biogenesis, and Plant Stress Responses

**DOI:** 10.3389/fpls.2012.00215

**Published:** 2012-09-17

**Authors:** Magdalena J. Mazur, Harrold A. van den Burg

**Affiliations:** ^1^Molecular Plant Pathology, Swammerdam Institute for Life Sciences, University of AmsterdamAmsterdam, Netherlands

**Keywords:** SUMO, chromatin, stress, heat shock, acetylation, histones

## Abstract

Small Ubiquitin-like MOdifier (SUMO) is a key regulator of abiotic stress, disease resistance, and development in plants. The identification of >350 plant SUMO targets has revealed many processes modulated by SUMO and potential consequences of SUMO on its targets. Importantly, highly related proteins are SUMO-modified in plants, yeast, and metazoans. Overlapping SUMO targets include heat-shock proteins (HSPs), transcription regulators, histones, histone-modifying enzymes, proteins involved in DNA damage repair, but also proteins involved in mRNA biogenesis and nucleo-cytoplasmic transport. Proteomics studies indicate key roles for SUMO in gene repression by controlling histone (de)acetylation activity at genomic loci. The responsible heavily sumoylated transcriptional repressor complexes are recruited by plant transcription factors (TFs) containing an (ERF)-associated Amphiphilic Repression (EAR) motif. These TFs are not necessarily themselves a SUMO target. Conversely, SUMO acetylation (Ac) prevents binding of downstream partners by blocking binding of their SUMO-interaction peptide motifs to Ac-SUMO. In addition, SUMO acetylation has emerged as a mechanism to recruit specifically bromodomains. Bromodomains are generally linked with gene activation. These findings strengthen the idea of a bi-directional sumo-acetylation switch in gene regulation. Quantitative proteomics has highlighted that global sumoylation provides a dynamic response to protein damage involving SUMO chain-mediated protein degradation, but also SUMO E3 ligase-dependent transcription of HSP genes. With these insights in SUMO function and novel technical advancements, we can now study SUMO dynamics in responses to (a)biotic stress in plants.

## Introduction

Over the last decade much has been learned on Small Ubiquitin like MOdifier (SUMO). SUMO is a ∼100 amino-acid polypeptide that is covalently attached to target proteins in a process closely resembling conjugation of the well-studied tag ubiquitin (Wilkinson and Henley, 2010; Park et al., 2011b). SUMO conjugation involves formation of an isopeptide bond between the C-terminal diglycine (diGly) residues of SUMO and the ε-amino group of lysines in target proteins. The machinery responsible for SUMO conjugation, including SUMO itself, is highly conserved and essential in many eukaryotes (Nacerddine et al., 2005; Saracco et al., 2007; Kaminsky et al., 2009). Hundreds of proteins have been identified as SUMO targets (e.g., Miller et al., 2010). SUMO conjugation affects these targets in different ways, such as (i) stability, (ii) sub-cellular localization (including recruitment to various nuclear foci), (iii) protein–protein interactions, and (iv) protein activity. Remarkably, the level of sumoylation detected on SUMO targets is often low with less than 10–20% modified. Yet, SUMO attachment appears to affect the function of the entire pool of a target protein; a phenomena termed the “SUMO enigma” (Wilkinson and Henley, 2010). Although the mechanisms are not fully understood, the notion is that sumoylation is sufficient to change target function by altering protein localization and protein–protein interactions, which apparently persist after SUMO deconjugation. For example, recruitment of histone deacetylases (HDAC) to promoters due to sumoylation of transcription factors (TFs) leads to promoter-specific histone deacetylation causing chromatin compacting, which favors transcriptional repression (Garcia-Dominguez and Reyes, 2009). Importantly, this compact chromatin structure apparently requires SUMO conjugation, but is largely independent of SUMO deconjugation.

**Figure 1:**
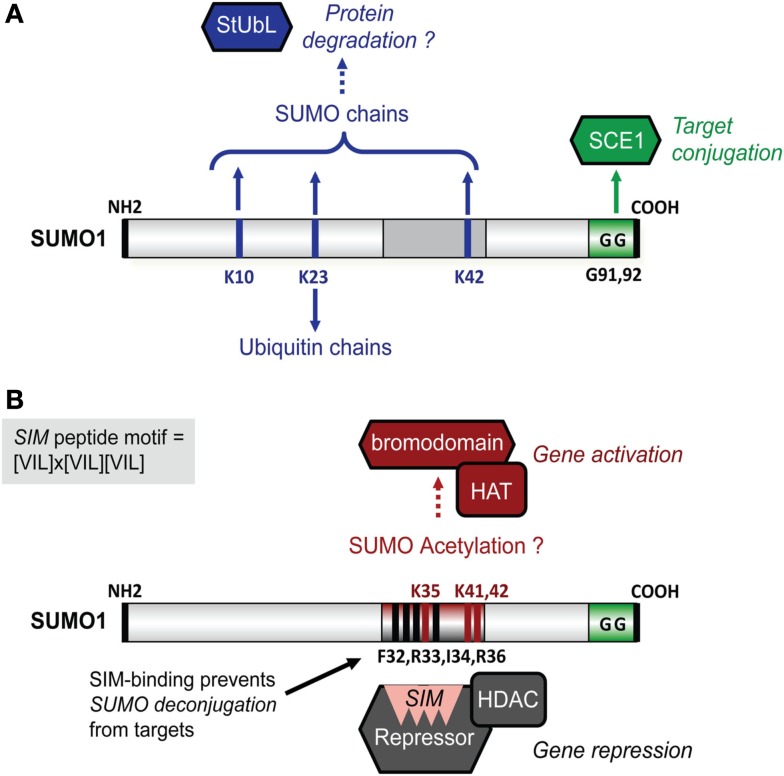
**Schematic structure of Arabidops SUMO1 and its potential interplay with other post-translational modifications**. **(A)** SUMO is conjugated to targets via SCE1 that forms an isopeptide with diGly residues (GG) of SUMO and Lys residues in target proteins. SUMO1 and SUMO2 (*not shown*) contain also internal acceptor sites for SUMO chain editing (Lys10, Lys23, and Lys42; *top*) and mixed ubiquitin-SUMO chains (Lys23; *bottom*). SUMO chains are recognized by StUbLs that conjugate ubiquitin on internal lysines in SUMO chains. This leads to 26S proteasome-mediated protein degradation of SUMO conjugates. **(B)** A SUMO-acetylation switch controls gene regulation by SUMO-modified targets. For example, SIM-dependent recruitment of co-repressor complexes is linked with (HDAC-mediated) gene repression (*bottom*). These SUMO-SIM interactions are disrupted by SUMO acetylation (*top*), which likely involves HAT activity. On the other hand, SUMO acetylation (on possibly Lys35, Lys41, and/or Lys42) allows SUMO instead to interact with bromodomains; a domain found in transcriptional co-activators. In addition, SUMO-SIM interactions appear to prevent SUMO deconjugation by SUMO proteases.

Critical for SUMO function is a binding pocket on SUMO that acts as a docking site for SUMO-interaction motifs (SIMs). This short peptide motif is found in partner proteins and comprises three hydrophobic residues that surround one additional residue (x), i.e., [VIL]x[VIL][VIL] or [VIL][VIL]x[VIL] (Kerscher, 2007; Figure 1). The SIM core aligns as an additional β-strand in the β-sheet of SUMO. In many cases, the SIM hydrophobic core is flanked by acidic residues (Asp/Glu) that provide additional electrostatic interactions with a basic interface on SUMO that surrounds the SIM-binding pocket. As SIM-containing partners are involved in a wide range of biological processes, it has proven to be difficult to predict the consequence of SUMO attachment for SUMO targets.

SUMO is commonly attached to Lys residues located in the consensus motif ΨKxE, where Ψ denotes a large hydrophobic residue (VILMFPC; Matic et al., 2010) and x represents any residue. This ΨKxE motif is recognized by the E2 SUMO conjugating enzyme SCE1 and this recognition is often sufficient for sumoylation (Bernier-Villamor et al., 2002). In fact, *in vitro* sumoylation reactions require usually only the E1 SUMO activating enzyme (SAE1/SAE2 dimer), SCE1, SUMO, and ATP. Proteomics studies have also identified divergent sumoylation motifs, such as the inverted consensus motif, the hydrophobic cluster sumoylation motif (HCSM), and extended versions like the phosphorylation-dependent sumoylation motifs (PDSM; Anckar and Sistonen, 2007; Blomster et al., 2010; Matic et al., 2010). The different motifs are frequently found in non-sumoylated proteins and are, therefore, not sufficient to predict SUMO targets. Conversely, sumoylation is also known to occur at non-consensus sites (between 20 and 40%). Together, this signifies that motif-based sequence searches with “known” sumoylation consensus motifs are not sufficient to unequivocally identify SUMO acceptor sites. To identify these sites, SUMO proteomics studies are needed.

## Approaches and Opportunities for Next Generation SUMO Proteomics

To perform SUMO proteomics, SUMO conjugates are now routinely purified using affinity-purification of His-tagged SUMO variants. While identification of the purified SUMO targets with mass spectrometry provides little problems, the identification of SUMO acceptor lysines in these targets remains difficult, as the MS/MS spectra corresponding to the modified isopeptides are often too complex to detect diGly-remnants or worse large SUMO tags left after tryptic digestion. In most cases, SUMO acceptor lysines are identified for each target separately using often MS/MS data obtained from *in vitro* sumoylated proteins. Such relatively simple MS/MS spectra are then analyzed with specific algorithms such as SUMmOn and ChopNSpice to facilitate annotation of both *in vitro* and biological data (Pedrioli et al., 2006; Hsiao et al., 2009; Jeram et al., 2010). A second problem is that tryptic digestion of SUMO leaves a large signature tag; this is now routinely circumvented by introducing an additional tryptic cleavage site (Arg residue) in SUMO directly adjacent to the diGly motif (+RGG C-terminus), which only leaves a diGly remnant on modified lysines after trypsin cleavage (Wohlschlegel et al., 2006; Miller et al., 2010; Vertegaal, 2011). Importantly, these His-tagged SUMO-RGG variants are fully functional in yeast, mammalian cells, and Arabidopsis.

A major development in SUMO proteomics is selective enrichment of diGly-modified peptides when isolating SUMO conjugates. This method is based on a His-tagged SUMO (RGG) variant in which all internal lysines are replaced for arginines allowing tailored protease digestion of SUMO conjugates (Matic et al., 2010). These Lys-deficient SUMO proteins are sensitive to trypsin but insensitive to Lys-C protease, which only cleaves after Lys residues. Lys-C digestion will, therefore, harness intact His-tagged SUMO proteins conjugated to Lys-C-generated peptides. These SUMO-modified isopeptides can effectively be purified using the His-tag. Trypsin digestion will subsequently yield diGly-modified signature peptides of the original SUMO conjugates. This approach identified 103 SUMO acceptor sites using HeLa cell cultures (Matic et al., 2010). However, one should be careful about substituting all lysines in SUMO, considering their importance for SIM docking, SUMO chain editing, and SUMO acetylation (see below).

Another key improvement is the development of monoclonal antibodies that recognize diGly-remnants left on isopeptides after trypsin digestion (Xu et al., 2010; Xu and Jaffrey, 2011). Immunoprecipitation with these antibodies followed by mass spectrometry-based diGly-remnant profiling provided 11,054 (Wagner et al., 2011), 9,957 (Emanuele et al., 2011), and >19,000 Ubiquitin-modified sites (Kim et al., 2011). Application of this antibody for SUMO proteomics in Arabidopsis is now feasible, i.e., one can perform diGly-remnant profiling in the *sumo1;sumo2* double mutant by complementing it with a His-tagged SUMO1-RGG variant. In combination with differential labeling techniques such as iTRAQ or 15N-isotope labeling, diGly-remnant profiling should provide a robust tool for quantitative SUMO proteomics under different stress conditions. One remaining complication is that trypsin digestion will also create diGly-remnants that originate from Ubiquitin and NEDD8 (RUB1/Related to ubiquitin 1 in Arabidopsis) modifications. In order to distinguish SUMO from these other modifications, Miller et al. (2010) introduced a four-residue footprint (+QTGG) in Arabidopsis SUMO1 expressing a HIS-tagged H89R variant, which proved to be fully functional.

The first decade of SUMO research revealed extensive roles for SUMO in plant development including meristem differentiation and floral induction, defense signaling via the hormone salicylic acid, and adaptation to diverse abiotic stresses such as heat stress, drought, and cold (Kurepa et al., 2003; Catala et al., 2007; Miura et al., 2007; Ishida et al., 2009; Castro et al., 2012). For the coming era, we see three major challenges for research on sumoylation in plants. First, data on the spatio-temporal dynamics of target sumoylation remains missing for SUMO-controlled processes in plants. This requires (relative) quantification of SUMO conjugates in different cell types and conditions. Such approaches have come within reach due to the development of *in vivo*-biotin labeling of specific nuclei combined with a purification method to obtain these labeled intact nuclei (Deal and Henikoff, 2011). A second challenge is to perform SUMO target profiling for SUMO E3 ligases like SIZ1 (SAP and MIZ-finger domain-containing protein 1) and SUMO proteases. For example, only several SIZ1-specific SUMO targets have been identified so far, such as Inducer of CBF expression 1 (ICE1) and Global TF group E3 (GTE3; Miura et al., 2007; Garcia-Dominguez et al., 2008), while (de-)sumoylation of hundreds of SUMO targets must happen in a controlled manner in cells. Last-but-not-least, genome sequencing has revealed that several plant species contain additional SUMO paralogs other than the canonical SUMO isoforms. Evolution of divergent non-canonical SUMO genes has repeatedly occurred, e.g., grasses (Poaceae) have a unique diSUMO-like SUMO paralog (Srilunchang et al., 2010), while in Brassicaceae four additional SUMO paralogs have emerged (Kurepa et al., 2003). These paralogs have possibly unique roles in plant development and signaling, as seen for Arabidopsis SUMO3 (van den Burg et al., 2010). *In planta* expression of mature variants (with their diGly C-terminus exposed) of non-canonical Arabidopsis SUMO paralogs indicated that these paralogs possibly have preferred SUMO targets (Budhiraja et al., 2009). However, the extent to which conjugation of these non-canonical paralogs occurs remains unresolved, as biochemical data suggested that Arabidopsis SUMO3 and -5 are poor substrates for SUMO protease maturation and the E1 enzyme (Castano-Miquel et al., 2011). In agreement, overexpression of both mature and conjugation-deficient variants of SUMO3 did not affect global SUMO1/2 conjugation levels, while overexpression of SUMO1 or -2 variants caused global sumoylation (van den Burg et al., 2010). Studies on SUMO paralogs in mammals established that they have their own preferred SIM partners (Zhu et al., 2009). This provokes the idea that the non-canonical paralogs might act to control interactions between the canonical SUMOs and their partners. Hence, identification of both paralog-specific targets and interactors is needed to fully comprehend SUMO gene evolution in plants.

## Related Proteins are Sumoylated in Arabidopsis and Other Eukaryotes

SUMO proteomics studies have identified in total >2,000 substrates in various organisms (Li et al., 2004; Panse et al., 2004; Vertegaal et al., 2004, 2006; Wohlschlegel et al., 2004; Denison et al., 2005; Hannich et al., 2005; Wykoff and O’shea, 2005; Ganesan et al., 2007; Golebiowski et al., 2009; Matafora et al., 2009; Westman et al., 2010; Galisson et al., 2011; Tatham et al., 2011). The consensus motif (ΨKxE) is significantly overrepresented in these different proteomics sets, e.g., on average more than 2 consensus motifs are found per human SUMO-2 target while the same motif is found only 0.6 times on average per protein in the entire human proteome (Golebiowski et al., 2009). In yeast alone, >500 SUMO (ScSmt3) conjugates were identified (Makhnevych et al., 2009). Furthermore, SUMO-affinity purifications and two-hybrid (Y2H) protein–protein interaction studies in yeast revealed another >250 SUMO-interacting proteins. A related study with *Drosophila* (*D. melanogaster*) cells also identified hundreds of SUMO targets and interactors (Nie et al., 2009), while for *Caenorhabditis elegans* ∼250 candidate SUMO targets have been identified (Kaminsky et al., 2009).

Miller et al. (2010) reported >350 SUMO1 targets in Arabidopsis. They used a strategy that restored sumoylation to endogenous levels complementing a lethal *sumo1;sumo2* mutant with a His-tagged genomic *SUMO1* clone fused to its own promoter. Using a stringent purification protocol, they obtained a high-confidence list of plant SUMO targets from plant extracts of this complemented line. Another plant study identified 238 candidate targets using the Arabidopsis SCE1 (148 interactors) and the SUMO protease ESD4 (Early in short days 4; 154 interactors) as Y2H baits (Elrouby and Coupland, 2010). Interestingly, a substantial set of these interactors was identified using ESD4 as bait. This appears to contradict with the fact that ESD4-like SUMO proteases preferentially recognize SUMO. Structural studies with Ulp1, a yeast homolog of ESD4, revealed that these proteases bind SUMO via two independent sites: (i) a catalytic site that forms a narrow tunnel trapping the diGly tail and (ii) an exosite that binds a distant epitope on the SUMO surface (Mossessova and Lima, 2000). Based on this, the “ESD4-interactome” most likely reflects Arabidopsis proteins that are efficiently sumoylated by the yeast SUMO machinery and this allows their interaction with ESD4. In support of this, 65 of the interactors identified were found with both ESD4 and SCE1 as Y2H bait. In a related study, SUMO from yeast (ScSmt3) was used as bait to identify SUMO targets (Hannich et al., 2005). In this case, the putative SUMO targets were confirmed by co-expressing Ubiquitin-like specific protease 1 (Ulp1) in yeast, which prevented reporter gene activation for SUMO targets but not for non-covalent interactors. In contrast, ESD4 interactors were still able to activate the reporter gene. This suggests that removal of ScSmt3 from SUMO conjugates by ESD4 is possibly less efficient than by Ulp1 and this could then allow reporter gene activation.

In addition, we searched the compiled Arabidopsis SUMOylome with the prevalent consensus peptide motif [VILMFPC]KxE (Matic et al., 2010). Seventy-one percent of the Arabidopsis SUMO targets identified by mass spectrometry contained this motif (Miller et al., 2010) and it was on average 2.15 times represented in these proteins. In contrast, only 52% of the SCE1/ESD4 interactors contained the motif, but those with the motif still had on average 1.95 motifs per protein (Elrouby and Coupland, 2010). This could signify that the Y2H set contains a considerable number of SUMO interactors rather than SUMO targets.

The large-scale studies in Arabidopsis have provided a list of SUMO targets for which in many cases related proteins were previously identified as SUMO target in metazoans, yeast, *Drosophila*, and/or *C. elegans* (Budhiraja et al., 2009; Elrouby and Coupland, 2010; Miller et al., 2010; Park et al., 2011a). The list of plant targets includes heat-shock proteins (HSPs), chromatin-associated proteins, and proteins involved in mRNA biogenesis. Based on this, a conserved role for SUMO is seen in chromatin-modifying complexes, histone acetylation, mRNA biogenesis, and possibly also in global sumoylation induced by cellular stress. Below, we further discuss this overlap in SUMO-controlled processes.

## Sumoylation of Conserved Subunits of Chromatin-Modifying Protein Complexes

**Figure 2:**
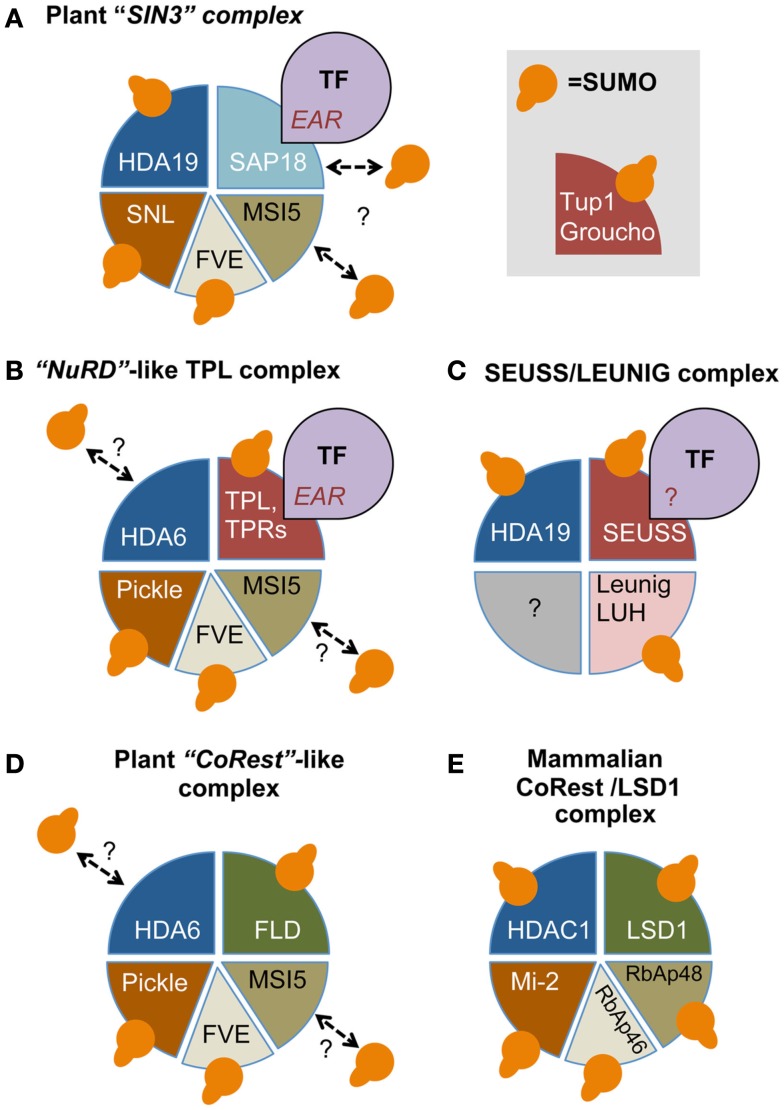
**Sumoylation impacts transcription repressor complexes conserved between mammals, yeast, and plants**. **(A)** The heavily sumoylated SIN3 co-repressor complex contains next to SNLs, AtSAP18, a class I histone deacetylase, e.g., HDA6/HDA19, and the histone-chaperones FVE and MSI5. Subunits for which sumoylation was established are indicated. **(B)** Likewise, the NuRD-like co-repressor complex contains – instead of SIN3/SAP18 – the SUMO targets Pickle (PKL; the plant homolog of the mammalian Mi-2) and the co-adaptor Topless (TPL) or its homologs (TPRs). The TPL/TPRs and SAP18 are both recruited to DNA-bound transcription factors (TFs) by EAR motifs present in these TFs. The homologs of TPL in other eukaryotes (Groucho/TUP1-like) are also sumoylated. **(C)** Another sumoylated repressor complex consists of the co-adaptors SEUSS and LEUNIG (LUG). The peptide motif involved in recruiting this complex is unknown (“?”). **(D)** Finally, the CoRest/LSD1-like complex that contains in addition the lysine demethylase Flowering locus D (FLD) is also sumoylated at several subunits. The mammalian homologs of the CoRest subunits are indicated in **(E)**. For further details on the role of SUMO in the different complexes we refer to the main text.

A comprehensive analysis of SUMO targets in yeast and mammals established that many conserved histone-modifying enzymes, their co-regulators, ATP-dependent nucleosome-remodeling proteins, and histone chaperones are SUMO-modified (Garcia-Dominguez and Reyes, 2009). These proteins are integral subunits of chromatin-modifying complexes that are largely conserved between eukaryotes. Upon recruitment of these complexes by specific DNA-binding TFs, they control the accessibility of DNA and concomitantly gene expression. Examples include the SWI-independent 3 (SIN3)-HDAC complex, the Nucleosome-Remodeling and histone Deacetylation (NuRD) complex, and the HDAC-containing CoRest/LSD1 (Lysine-specific demethylase 1) repressor complex. Subunits of these complexes are also sumoylated in Arabidopsis including multiple members of various protein families. For example, different subunits of the Arabidopsis the SIN3 complex are sumoylated including SIN3-like homolog 2 (SNL2), SNL4, SNL5, and the class I histone deacetylase HDA19 (Figure 2A; Song et al., 2005; Song and Galbraith, 2006; Miller et al., 2010). Sumoylation of class I HDACs other than HDA19 has not yet been shown. Another putative SUMO target in the SIN3 complex is the subunit AtSAP18 (SIn3-associated protein, 18kDA), as sumoylation of HsSAP18 was established in HeLa cells (Golebiowski et al., 2009).

Besides the SIN3 complex, the NuRD complex also appears to be heavily sumoylated (McDonel et al., 2009; Figure 2B). The core of this complex is formed by the aforementioned class I HDACs and the ATPase nucleosome-remodeling factor Mi-2, which is also a SUMO target in HeLa cells (Golebiowski et al., 2009). The Arabidopsis homologs of Mi-2 are PKL (Pickle) and Pickle related 1 (PKR1, Chromatin Remodeling 4), which both were SUMO-modified *in planta* (Aichinger et al., 2009; Miller et al., 2010; Zhang et al., 2012). In support of a function of PKL/PKR1 in transcriptional repression, PKL appears to associate physically with histone H3K27 trimethylation (H3K27me3) enriched regions, a mark for gene silencing.

Studies on PKL/PKR1 homologs indicate that they might also be directly recruited to sumoylated TFs and this is possibly independent of the NuRD complex or HDAC activity. For example, Mi-2 is also part of the HDAC1-independent repressor complex *Drosophila* MEP-1-containing complex (dMec), as shown by studies on the *Drosophila* TFs Sp3 and Dorsal (Kunert and Brehm, 2009; Kunert et al., 2009). Recruitment of dMi-2 to Sp3 and Dorsal requires sumoylation of these TFs (Stielow et al., 2008a,b, 2010). However, silencing of NuRD subunits other than Mi-2 did not affect gene repression, while silencing of dMec components lifted sumoylation-dependent transcriptional repression. Also treatment with HDAC inhibitors did not influence Sp3-SUMO-mediated gene repression. Clearly, *Drosophila* dMi-2 can also trigger gene repression in an HDAC-independent manner, which involves apparently prior sumoylation of DNA-bound TFs.

In addition to HDACs, the NuRd and SIN3 repressor complexes also share the SUMO targets RbAp46 and RbAp48 that act as histone chaperones and bind the histone dimer H3–H4 (Murzina et al., 2008). In Arabidopsis, these chaperones are represented by five homologs, including FVE/Multicopy Suppressor of IRA1 4 (MSI4) and MSI5 (Ausin et al., 2004; Jeon and Kim, 2011). So far, sumoylation has only been shown for FVE (Miller et al., 2010). These histone chaperones are in fact part of many chromatin-modifying complexes including a complex with histone acetyltransferase (HAT1), the Chromatin Assembly Factor-1 (CAF-1) that deposits nucleosomes, the Polycomb Repressive-like Complex 2 (PRC2) that catalyzes histone H3K27me3 (Loyola and Almouzni, 2004), and the CoRest/LSD1 repressor complex (Baron and Vellore, 2012; Figures 2D,E). LSD1 is, however, also reported to be part of the NuRD complex (Wang et al., 2009). Clearly, the NuRD and the CoRest/LSD1 complexes share many subunits. For example, physical interactions have been shown between Arabidopsis FVE, MSI5, HDA6, and Flowering locus D (FLD), which is an Arabidopsis homolog of LSD1 (Gu et al., 2011; Jeon and Kim, 2011; Yu et al., 2011). In addition, genetic data indicated that both FVE and FLD are required for HDA6-mediated deacetylation of the target locus Flowering locus C (FLC; He et al., 2003; Kim et al., 2004). Importantly, FLD acts as a SUMO target and sumoylation of FLD appears to suppress its repressor function, since expression of a sumoylation-deficient mutant of FLD in *fld* protoplasts strongly reduced *FLC* expression in comparison to wild type FLD (Jin et al., 2008).

It is too early to draw general conclusions on the effect of SUMO on HDAC recruitment and its activity when recruited to plant transcriptional repressor complexes like NuRD or SIN3. For example, sumoylation of human HDAC1 enhanced its transcriptional repression (David et al., 2002), while recruitment of *Drosophila* HDAC1 to sumoylated TFs depends on a SIM in HDAC1 itself, as shown for the *Drosophila* co-repressor Gro (Groucho; Ahn et al., 2009). Conversely, sumoylation of certain targets leads to displacement of HDACs from these targets, like for CoRest (Gocke and Yu, 2008).

SUMO not only controls HDAC activity in plants, but also histone demethylase, HAT, and histone methyltransferase (HMT) activity. For example, the HAT GCN5 and its two adaptor proteins Ada2a (transcriptional ADAptor 2a) and Ada2b are SUMO targets (Miller et al., 2010; Servet et al., 2010). The Ada2 adaptors enhance HAT activity of General control of amino-acid synthesis 5 (GCN5) and recruit GCN5 to TFs (Mao et al., 2006; Samara and Wolberger, 2011). Together they are part of the larger SAGA-type HAT complex, which is largely conserved between yeast, *Drosophila*, and mammals. Proteomics studies in the latter two organisms showed that the SAGA complex is also sumoylated at various subunits (Golebiowski et al., 2009; Makhnevych et al., 2009). In yeast, Gcn5 sumoylation appears to inhibit SAGA-mediated gene expression (Sterner et al., 2006), which agrees with the notion that sumoylation generally causes gene repression. In Arabidopsis, GCN5 is associated with about one third of 20,000 promoter regions analyzed (Benhamed et al., 2008). This means that regulation of histone (de)acetylation by SUMO could be widespread in Arabidopsis, involving many transcriptional programs at various genomic loci.

## SUMO Controls TOPLESS and Other Plant Co-Adaptors Involved in Gene Repression

An important co-adaptor family is formed by the Groucho/Transducin 1-like (Gro/Tup1-like) family that mediates gene repression by acting with HDACs (Jennings and Ish-Horowicz, 2008; Ahn et al., 2009). The Arabidopsis genome encodes 14 Gro/Tup1-like co-adaptors (Liu and Karmarkar, 2008; Lee and Golz, 2012). Many of these Gro/Tup1-like homologs are sumoylated *in planta* including Leunig (LUG), LUG homolog (LUH), Topless (TPL), and TPL-related proteins 1 to 4 (TPR1, -2, -3, -4; Miller et al., 2010; Figures 2B,C). The TPL/TPRs appear to be part of a NuRD-like protein complex (Figure 2B). At least TPL interacts with FVE and PKR1, while FVE and MSI5 associate with HDA6 (Gu et al., 2011; Causier et al., 2012). Based on their domain organization, LUG/LUH form a different class than TPL/TPRs. LUG and LUH act redundantly and both interact physically with HDA19 and another co-adaptor SEUSS that recruits them to DNA-binding TFs (Sridhar et al., 2004; Gonzalez et al., 2007; Figure 2C). Also SEUSS is a SUMO target *in planta* and its SUMO acceptor site has been identified (VK^200^xE; Miller et al., 2010). This site is conserved in three of the four Arabidopsis SEUSS homologs (SEU, SLK2, and SLK3). SUMO acceptor sites have not yet been identified for TPL or the other TPRs.

The consequence of SUMO attachment is unknown for both TPL/TPRs and LUG/SEU, but it has been studied for their *Drosophila* homolog Gro. SUMO conjugation of Gro promotes its transcriptional repressor activity via enhanced recruitment of HDAC1 (Ahn et al., 2009). An alternative mechanism emerged from studies of the *Drosophila* SUMO-targeted Ubiquitin ligase (StUbL) Degringolade (Dgrn). StUbLs bind poly-SUMO chains and target poly-SUMO-modified conjugates for proteasomal degradation by attaching ubiquitin to lysines in the SUMO chain (Perry et al., 2008; Denuc and Marfany, 2010). Dgrn binds to poly-sumoylated Gro and lifts Gro-mediated transcriptional repression. Dgrn does not bind to Gro itself but rather to the associated TF Hairy, which recruits Gro and the SUMO chains attached to Gro (Abed et al., 2011). These authors proposed that sumoylation sequestered Gro in larger oligomers. This antagonism between Drgn and Gro was not Hairy-specific, but affected Hairy-independent loci as well. In a similar manner, plant StUbLs might lift TPL/TPRs- or LUG/SEU-based gene repression sequestering them in larger oligomers.

## EAR-Containing TFs are Not *per se* SUMO Targets Despite Their Role in Gene Repression

The TPL/TPRs interactome has recently been exposed using Y2H approaches, revealing >200 partners including many known interactors (Arabidopsis Interactome Mapping Consortium, 2011; Causier et al., 2012). This interactome included a wide range of TFs, many of which were previously implicated in transcriptional repression. Importantly, TPL/TPRs lack a clear DNA-binding domain. Instead, they are recruited to specific TFs that contain an Ethylene-responsive element binding factor (ERF)-associated Amphiphilic Repression (EAR) motif (Kagale and Rozwadowski, 2011; Causier et al., 2012). Considering the close ties between SUMO and HDAC repressor complexes, we examined the extent to which EAR-containing TFs are also subject to sumoylation, as their sumoylation might influence recruitment of co-adaptors or other proteins to repressor complexes. We noted only a small overlap (27 proteins) between the TPL/TPRs interactome and the list of Arabidopsis SUMO substrates identified (Table A1 in Appendix). Hence, EAR- and sumoylation-mediated recruitment of chromatin-modifying complexes to TFs likely involves different sets of TFs. Consequently, EAR-dependent recruitment of co-adaptors like TPL and AtSAP18 likely does not require sumoylation of the TFs involved. A similar situation was reported for *Drosophila*; the SUMO consensus motif was enriched in *Drosophila* TFs with a dual function in gene regulation (both induction and gene repression), while it was not in TFs that were predicted to have a single activity (Bauer et al., 2010).

## SUMO Acetylation Blocks SUMO-SIM Interactions and Promotes Bromodomain-Dependent Gene Activation

While SUMO modifies HDAC- and HAT-containing complexes, SUMO itself is a substrate for acetylation. SUMO acetylation (Ac) has been reported to mimic acetylation of the TF modified by Ac-SUMO (Cheema et al., 2010). SUMO acetylation neutralizes the basic charges that surround the SIM docking site and, most remarkably, prevents SUMO-SIM interactions (Ullmann et al., 2012; Figure 1B). Possibly, SUMO acetylation acts as a first step to resolve SUMO-mediated protein interactions, because when bound to SIMs certain SUMO conjugates are protected from deconjugation (Zhu et al., 2009). SUMO acetylation does not only attenuate “SIM-SUMO”-dependent gene silencing, but it also promotes SUMO-bromodomain interactions (Ullmann et al., 2012). Bromodomains are typically found in transcriptional co-activators and are unique in that they bind acetylated histones (Mujtaba et al., 2007). Conversely, the plant homeodomain (PHD) domain present in the Arabidopsis SUMO E3 ligase SIZ1 is required for sumoylation of two bromodomain-containing TFs, GTE3, and GTE5; sumoylation of GTE3 suppressed its binding to acetylated histone H3 (Garcia-Dominguez et al., 2008). Similarly, the PHD domain in the mammalian co-repressor Krüppel-associated protein (KAP1) controls intramolecular sumoylation of the adjacent bromodomain required for KAP1-mediated gene silencing (Ivanov et al., 2007). In this case, the PHD domain acts as a specific SUMO E3 ligase for bromodomains. Moreover, class II HDACs have been reported to promote sumoylation of specific substrates, suggesting that they also act as SUMO E3 ligases (Garcia-Dominguez and Reyes, 2009), while HATs likely promote SUMO acetylation. These findings support a model in which “PHD domain-mediated bromodomain sumoylation” and “HDAC/HAT-mediated SUMO (de)acetylation” provide the cell with a bi-directional transcriptional switch involving “SUMO-SIM” dependent gene silencing and “AcetylSUMO-bromodomain” dependent gene activation, respectively (Figure 1B).

## Sumoylation Controls mRNA Biogenesis and Nuclear Export

Small-Ubiquitin-like MOdifier proteomics studies have also revealed a major role for SUMO in mRNA biogenesis including mRNA processing, editing, and nuclear export in different eukaryotes including plants, as recently reviewed (Vethantham and Manley, 2009; Meier, 2012). The notion is that transient sumoylation events in the nucleus form a critical step in mRNA surveillance to retain unspliced pre-mRNAs in the nucleus. Currently, 39 Arabidopsis SUMO targets have been identified with a confirmed or predicted role in mRNA biogenesis. Studies in yeast and mammalian cells also revealed SUMO targets involved in 5′ pre-mRNA capping, splicing, 3′ processing, and mRNA export (Vethantham and Manley, 2009). For example, small nuclear ribonucleoproteins (snRNPs) involved in splicing of pre-mRNA in the spliceosome are SUMO targets. Moreover, several heterogeneous nuclear ribonucleoproteins (hnRNPs) that bind pre-mRNA are SUMO targets (Li et al., 2004; Blomster et al., 2009). Sumoylation of hnRNP C and M decreases their binding to nucleic acids (Vassileva and Matunis, 2004).

Notably, components of the plant nuclear pore complex (NPC) such as Importin-6 (IMP-6), IMPα1, and WPP domain interacting protein 1 (WIP1) are SUMO targets (Miller et al., 2010). Arabidopsis Nuclear pore anchor (NUA) is also a SUMO target, at least *in vitro* (Elrouby and Coupland, 2010). NUA interacts physically with the SUMO protease ESD4 at the nuclear rim (Xu et al., 2007). Loss of function mutations in ESD4, SIZ1, but also of two Arabidopsis genes involved in mRNA trafficking, NUA, and the scaffold nucleoporin Nup160, resulted in nuclear retention of both SUMO conjugates and mRNA (Xu et al., 2007; Muthuswamy and Meier, 2011). In addition, ESD4, its yeast homolog Ulp1, and its mammalian homologs SENP1 (Sentrin-specific protease 1) and SENP2 all localize to the inner side of the nuclear envelope through association with NPCs (Murtas et al., 2003; Mukhopadhyay and Dasso, 2007; Xu et al., 2007). Hence, Arabidopsis NUA and Nup160 connect SUMO conjugation directly with general nuclear import and export via NPCs in plants. Overall, the emerging picture is that SUMO controls many steps in mRNA biogenesis and nucleo-cytoplasmic trafficking.

## Global (Poly-)Sumoylation as Response to Stress-Induced Protein Damage

When exposed to abiotic stresses, such as heat shock, drought, or freezing, plants respond with global protein sumoylation (Kurepa et al., 2003). Similarly, protein-damaging agents like ethanol or the non-protein amino acid l-canavanine trigger global SUMO conjugation in Arabidopsis (Kurepa et al., 2003). This is clearly a general response, which is also seen in yeast and mammalian cells in response to heat stress and protein damage and it is essential for cell survival of HeLa cells after heat stress (Saitoh and Hinchey, 2000; Golebiowski et al., 2009; Tatham et al., 2011). Quantitative proteomics studies on HsSUMO-2 conjugation in HeLa cells have revealed that heat stress triggers differential sumoylation of hundreds of proteins (Golebiowski et al., 2009). Interestingly, many subunits of repressor complexes including SIN3, NuRd, and SetDB1 (that methylates histones which in turn promotes binding of HP1 proteins to maintain chromatin silencing) complexes showed enhanced sumoylation upon heat stress in HeLa cells. In contrast, sumoylation of the histones H2A, H2B, and H4, but also HDACs and HATs was reduced upon heat shock in these cells. Hence, many subunits of chromatin remodeling complexes become sumoylated, while the responsible enzymes and histones are deSUMOylated. Similar changes in Arabidopsis SUMO1 conjugation levels were reported for specific groups of proteins when seedlings were exposed to heat stress (Miller and Vierstra, 2011). For example, the co-adaptors TPL, SEU, PKL, SWI3C, and CHR11 were more sumoylated upon heat stress. On the other hand, Arabidopsis histone H2B was less sumoylated after heat stress, as seen for HeLa cells.

One consequence of heat stress is that RNA splicing is generally inhibited (Yost and Lindquist, 1986). Concomitantly, snRNPS involved in RNA splicing showed less sumoylation upon heat stress in mammalian cells. Splicing inhibition promotes production and export of mRNAs coding for HSPs, as the corresponding genes generally lack introns, thereby allowing HSP-mediated cellular recovery after heat stress (Golebiowski et al., 2009). A follow-up proteomics study revealed that sumoylation levels changed for 564 out of 1355 HsSUMO-2 targets when the 26S proteasome was inhibited with MG132 (Tatham et al., 2011). Interestingly, the global sumoylation response triggered by heat stress is positively correlated with the response triggered by proteasome inhibition involving largely overlapping sets of targets. However, inhibition of protein synthesis blocked the global sumoylation response induced by proteasome inhibition, but not by heat stress. This implies that newly synthesized misfolded SUMO targets are destined for protein degradation, while heat stress enhances sumoylation of existing proteins possibly to aid their refolding (also in this case the responses are correlated, i.e., largely the same proteins are sumoylated; Tatham et al., 2011).

The picture is even more complex, as heat stress also triggered poly-SUMO chain editing on >900 SUMO targets in HeLa cells (Bruderer et al., 2011). SUMO targets implicated in gene regulation and chromatin structure were almost exclusively modified with five or more SUMO molecules, while SUMO targets involved in DNA replication, DNA repair, and mRNA biogenesis had variable SUMO chain lengths starting from three. Other studies indicated earlier that ubiquitin only co-purifies with SUMO isoforms that contain an internal acceptor site utilized for poly-SUMO chain formation, but not with isoforms that lack such sites like HsSUMO-1 (Schimmel et al., 2008). These poly-SUMO chains serve as docking site for StUbLs (Perry et al., 2008; Denuc and Marfany, 2010). These findings demonstrate an unexpected large regulatory role for StUbL-dependent protein degradation of SUMO conjugates after heat stress. Also in plants poly-SUMO chains and mixed ubiquitin-SUMO chains have been found (Miller et al., 2010). This means that poly-SUMO chain-mediated protein degradation likely occurs in plants. In support, *in vitro* sumoylation assays indicated that the canonical Arabidopsis SUMO isoforms SUMO1 and -2 contain an internal SUMO acceptor site (Lys10) used for SUMO chain formation (Colby et al., 2006; Figure 1A). Interestingly, *in vivo* studies revealed SUMO chain editing on other SUMO1 residues (Lys23 and Lys41), while Lys23 was also subject to ubiquitination (Miller et al., 2010). StUbLs contain tandem arrayed SIMs in their N-termini that recognize poly-SUMO chain-modified proteins and RING-finger domains in their C-termini involved in ubiquitination. Based on this, a putative StUbL protein family was recently proposed for plants based on protein sequence homology and sequence conservation across different plant species (Novatchkova et al., 2012). However, data on the function of this potential Arabidopsis StUbL is missing.

Within 5 min after heat stress, the mammalian SUMO machinery, including the SIZ1 homolog PIASy and the SUMO conjugating enzyme Ubc9, are transiently recruited to the *HSP70.1* promoter and induce PIASy-dependent sumoylation of PARP-1 [Poly(ADP-ribose) polymerase 1; Martin et al., 2009; Messner et al., 2009]. Poly(ADP-ribose) is associated with chromatin decompacting and nucleosome loss. PIASy-mediated sumoylation of PARP-1 is necessary for full activation of the *HSP70.1* gene. Martin et al. proposed that heat shock induces rapid sumoylation of PARP-1 at the *HSP70.1* promoter, followed by ubiquitylation and degradation. The latter requires the StUbL RNF4 (RING-finger protein 4) suggesting that PARP-1 is modified with poly-SUMO chains. In Arabidopsis, AtPARP1 becomes also sumoylated upon heat stress (Miller et al., 2010). Therefore, the effect of SUMO on PARP-1 function in heat stress is likely conserved in Arabidopsis. Moreover, overexpression of cytosolic HSP70 enhanced heat tolerance in Arabidopsis seedlings, while suppressing the global SUMOylation responses triggered by heat stress (Kurepa et al., 2003). These data, thus, suggest that heat shock-dependent PARP-1 sumoylation and degradation increases *HSP70* mRNA levels and correspondingly its protein levels, while a 4–5 fold increase in HSP70 protein levels suppresses the global sumoylation response in Arabidopsis.

Overall, these findings imply that sumoylation controls heat-shock responses at (i) the level of *HSP* transcripts, (ii) pre-mRNA processing level favoring nuclear export of *HSP* transcripts, and (iii) at the level of protein folding and degradation of misfolded proteins, until the levels of HSPs have increased to sufficient levels to deal with protein damage caused by heat stress.

## Conflict of Interest Statement

The authors declare that the research was conducted in the absence of any commercial or financial relationships that could be construed as a potential conflict of interest.

## Appendix

**Table A1:** **The overlap between the SUMO interactome and proteins that interact with topless (TPL) and TPL-related 1–4 from Arabidopsis**.

**AGI code**	**Gene name**	**Annotation**
At1g07310	–	Calcium-dependent lipid-binding (CaLB domain) family protein
At1g10760	SEX1	STARCH EXCESS 1; Encodes an #945;-glucan, water dikinase required for starch degradation. Involved in cold-induced freezing tolerance.
At1g23190	PGM3	Cytosolic phosphoglucomutase (PGM
At1g43170	RP1	RIBOSOMAL PROTEIN 1 (RP1)
At1g62300	WRKY6	Regulates Phosphate1 (Pho1) expression in response to low phosphate (Pi) stress
At1g67090	RBCS1A	Ribulose bisphosphate carboxylase small chain 1A
At2g01350	QPT	QUINOLINATE PHOSHORIBOSYLTRANSFERASE, involved in NAD biosynthesis
At2g19520	FVE (MSI4)	Homolog of the mammalian retinoblastoma-associated protein (RbAp), one component of a histone deacetylase (HDAC) complex involved in transcriptional repressionControls flowering. protein_coding (FVE); (ACG1); (ATMSI4); (NFC4); (NFC04);MULTICOPY SUPPRESSOR OF IRA1 4 (MSI4)
At2g45640	SAP18	Interacts with SIN3, HDA19/HDA6 co-repressors complex
At3g01090	AKIN10	NF1-related protein kinase that physically interacts with SCF subunit SKP1/ASK1 and 20S proteosome subunit PAD1. It can also interact with PRL1 DWD-containing protein. Based on *in vitro* degradation assays and cul4cs and prl1 mutants, there is evidence that AKIN10 is degraded in a proteasome-dependent manner, and that this depends on a CUL4-PRL1 E3 ligase protein_coding SNF1 KINASE HOMOLOG 10 (KIN10) SNF1 KINASE HOMOLOG 10 (AKIN10);SNF1 KINASE HOMOLOG 10 (KIN10); (KIN10)
At3g02550	AS2	Asymmetric leaves 2, LOB domain-containing protein 41 (LBD41); CONTAINS InterPro DOMAIN
At3g10390	FLD	SWIRM domain-containing protein found in histone deacetylase complexes in mammals
At3g10480	NAC050	Transcription factor
At3g10490	NAC052	Transcription factor
At3g13920	EIF4A1	Eukaryotic translation initiation factor 4A-1
At3g16500	IAA26 (PAP1)	Phytochrome-associated protein 1
At3g17900	Unknown	Gene of unknown function
At3g20770	EIN3	Ethylene-insensitive3), a nuclear transcription factor that initiates downstream transcriptional cascades for ethylene responses
At3g52250	–	Protein with a putative role in mRNA splicing.
At4g11660	AT-HSFB2B	HEAT-SHOCK TRANSCRIPTION FACTOR B2B
At4g17330	unknown	Gene of unknown function
At4g29130	HXK1	GLUCOSE INSENSITIVE 2 (GIN2);HEXOKINASE 1 (HXK1); Functions as a glucose sensor to interrelate nutrient, light, and hormone signaling networks for controlling growth and development in response to the changing environment.
At5g28540	BIP	Luminal binding protein BiP, an ER-localized member of the HSP70 family
At5g42020	BIP2	Luminal binding protein (BiP2) involved in polar nuclei fusion during proliferation of endosperm nucle
At5g43700	IAA4	INDOLE-3-ACETIC ACID INDUCIBLE 4 (IAA4);AUXIN INDUCIBLE 2–11 (ATAUX2–11)
At5g44800	CHR4 (PKR1)	Chromatin remodeling 4 (CHR4), Pickle related 1
At5g55070	–	Dihydrolipoamide succinyltransferase;
At5g66140	PAD2	PROTEASOME ALPHA SUBUNIT D2 (PAD2); Encodes alpha5 subunit of 20S proteosome complex involved in protein degradation
